# Estimation of Aortic Stiffness with Bramwell–Hill Equation: A Comparative Analysis with Carotid–Femoral Pulse Wave Velocity

**DOI:** 10.3390/bioengineering9070265

**Published:** 2022-06-21

**Authors:** Luca Mesin, Luca Floris, Piero Policastro, Stefano Albani, Paolo Scacciatella, Nicola Pugliese, Stefano Masi, Andrea Grillo, Bruno Fabris, Francesco Antonini-Canterin

**Affiliations:** 1 Mathematical Biology and Physiology, Department Electronics and Telecommunications, Politecnico di Torino, 10129 Turin, Italy; luca.floris@studenti.polito.it (L.F.); piero.policastro@polito.it (P.P.); 2 Division of Cardiology, Umberto Parini Regional Hospital, 11100 Aosta, Italy; albani.aosta@gmail.com (S.A.); pscacciatella@ausl.vda.it (P.S.); 3 Department of Clinical and Experimental Medicine, University of Pisa, 56126 Pisa, Italy; n.r.pugliese88@gmail.com (N.P.); stefano.masi@unipi.it (S.M.); 4 Department of Medicine, Surgery and Health Sciences, University of Trieste, 34149 Trieste, Italy; andr.grillo@gmail.com (A.G.); b.fabris@fmc.units.it (B.F.); 5 Highly Specialized in Rehabilitation Hospital—ORAS S.p.A., 31045 Motta di Livenza, Italy; francesco.antoninicanterin@ospedalemotta.it

**Keywords:** aorta, stiffness, Bramwell–Hill equation, pulse wave velocity, ultrasound

## Abstract

Aortic stiffness is an important clinical parameter for predicting cardiovascular events. Carotid–femoral pulse wave velocity (cf-PWV) has been proposed for performing this evaluation non-invasively; however, it requires dedicated equipment and experienced operators. We explored the possibility of measuring aortic stiffness using ultrasound scans of the abdominal aorta coupled with the Bramwell–Hill equation. Healthy subjects were investigated; measurements of cf-PWV were taken by arterial tonometry and aortic systo-diastolic pressure difference was estimated using a validated model. Pulsatility of an abdominal tract of aorta was assessed by automated processing of ultrasound scans. Through a Bland–Altmann analysis, we found large biases when estimating each parameter by applying the Bramwell–Hill equation to the measured values of the other two paramters (bias, ± 1.96 SD; PWV, about 2.1 ± 2.5 m/s; pulsatility, 12 ± 14%; pressure jump, 47 ± 55 mmHg). These results indicate that the two measures are not interchangeable, and that a large part of the bias is attributable to blood pressure estimation. Further studies are needed to identify the possible sources of bias between cf-PWV and aortic pulsatility.

## 1. Introduction

The loss of the cushioning function of large arteries leads to the development of arterial stiffness, which occurs with aging and various pathological states [1]. The stiffening of large arteries, in particular the aorta, has important hemodynamic consequences, leading to systolic hypertension, organ damage in the kidneys and brain, and myocardial remodelling and consequent heart dysfunction [2]. The evaluation of aortic stiffness has been widely validated as a clinical predictor of cardiovascular morbidity and mortality, with a predictive value independent of conventional risk factors. Nevertheless, arterial stiffness measurement has yet to be included in routine clinical practice, possibly due to methodological issues and the variety of methods that have been proposed [3].

Measuring the stiffness (i.e., the reciprocal of distensibility) of an artery non-invasively has been a technical issue for a long time [4]. Evaluation of pulse wave velocity (PWV), i.e., the speed of a pulsation along a vessel, has been widely recognized as the most practical method of measurement in humans, providing the average stiffness of a vessel over a segment [5]. The stiffness of the aorta, the most important vessel to consider in the clinical setting, is conventionally measured by carotid–femoral pulse wave velocity (cf-PWV), which requires dedicated equipment in the form of pressure sensors (tonometers) [6]. This non-invasive parameter has been widely validated both clinically and instrumentally against invasive measurements of aortic PWV, showing high reliability [7].

The Bramwell-Hill equation [8] provides the relationship between arterial distensibility and PWV, which that indicates that arterial stiffness can be fully defined by the relationship between arterial pressure and the cross-sectional area of the vessel. In the physiological pressure ranges that occur during the cardiac cycle, the distensibility can be calculated by determining the fluctuations from diastole to systole over certain local pressures and areas. A single value of the local PWV in a vessel can thus be obtained by applying these data within the Bramwell–Hill equation.

Ultrasounds are commonly used to explore arterial morphology in the clinical setting. Arterial cross-sectional area changes, i.e., from systole to diastole, are considered in order to calculate local PWV values, especially in the carotid [9,10] and femoral arteries [11]; more recently, a similar approach has been suggested in the aorta [12]. These measures provide a value of the local PWV in a cross-section of the vessel from the diameters and values of blood pressure and blood density, which may differ from measures of PWV (such as cf-PWV). In fact, an error in estimating blood pressure, blood density, or vascular sizes may be reflected in a bias in the estimate of local PWV; vice versa, the measurement of PWV might not indicate precisely the variations in blood pressure or the size of the investigated vessel.

The aim of our study was to investigate this problem, focusing on measurements of aortic stiffness, PWV, and blood pressure and their relationship as provided by the Bramwell–Hill equation. Pulsatility in the abdominal aorta was obtained by ultrasound evaluation, cf-PWV was measured with arterial tonometers, and blood pressure was computed by validated models. Then, these values were determined using the Bramwell–Hill equation applied to each pair of variables to estimate the third, and possible sources of bias between the measurements and the estimations were investigated.

## 2. Methods

### 2.1. Carotid–Femoral Pulse Wave Velocity

Carotid–femoral PWV (cf-PWV) was measured non-invasively by tonometry with the foot to foot method, which represents the gold standard. The foot of the curve corresponds to the end-diastole point as identified by the tangent method [6].

Two different instruments were used to measure the delay (Δt) of the two pressure waves recorded at the carotid and femoral arteries: the PulsePen® device (Diatecne Srl., Milan, Italy), used by the team working in Trieste, and the SphygmoCor® (AtCor medical Pty Ltd., Sydney, Australia), used by Pisa group.

With the PulsePen® device, composed of one tonometer and an integrated electrocardiogram unit, the delay was calculated in two steps: the operator first simultaneously recorded the pulse wave with the probe placed over the carotid and the ECG; then, he repeated the procedure placing the probe in the femoral artery. The delay (Δt), was then computed as the difference between the delays between the pulse wave at the level of the carotid or femoral artery and the R wave of the ECG.

The SphygmoCor® device has two probes, enabling the operator to simultaneously record the carotid and femoral artery pulse waves; the delay (Δt) was defined as the difference between the two feet of the waves.

With the distance (ΔL) between the carotid and femoral arteries, the PWV can be calculated as

(1) $$PWV=\frac{\Delta L}{\Delta t}$$

The measurement of the distance ΔL was carried out with a tape. The actual distance ΔL used in the above formula is 80% of the measured one. This is derived from the subtractive method, which takes into account the fact that the pressure wave, while travelling to the carotid artery, covers the same path along the aortic arch. This value was proposed as the most representative of the real distance between the carotid and femoral artery [6].

The measurement of the delay (Δt) was repeated for about ten heartbeats and then averaged to obtain a stable estimation.

### 2.2. Ultrasound

Ultrasound measurements were performed close to tonometry in order to obtain comparable data and reduce the bias caused by short-time blood pressure variations.

The following ultrasound systems were used: iE33®, Philips medical systems (Trieste group), and Lisendo 880®, Hitachi (Pisa group).

A long axis section of the abdominal aorta was visualized by subcostal view, following current recommendations [13]. The borders of the aorta were identified automatically with the algorithm proposed in [14] and used in different applications on the inferior vena cava [15,16,17,18]. In the first frame of the ultrasound video, the user indicated different information for the processing, e.g., the region of interest and two reference points to be tracked to compensate for possible movements and deformations of the artery. Each frame was pre-processed with a 2D median filter (in the neighborhood of 9 × 9 pixels). Then, the software uniformly distributed 21 lines in the region of interest (identified after compensating for possible movements) and identified the borders of the aorta along these lines as a jump in the intensity of the ultrasound.

After the two borders of the aorta were obtained, the software computed the mean diameter, $D_{m}\left(t\right)$, for each frame (recorded at time *t*) as the mean of five diameters uniformly distributed in the region of interest and placed orthogonally to the midline of the blood vessel [14]. From the mean diameter, the cross-section area was computed as

(2) $$A\left(t\right)={\left(\frac{D_{m}\left(t\right)}{2}\right)}^{2}\pi$$

Pulsatility was then measured from the computed area of the region of interest. Specifically, the dilation of the vessel during systole depends on its elasticity, which is predominantly determined by the elastin/collagen ratio. Pulsatility is a parameter that measures this dilation. It was computed as ΔA/A, where ΔA is the difference between the maximum and minimum value during a cardiac cycle and *A* is the mean area during the same time interval. Different heartbeats were included in the ultrasound scans (which lasted about 10 s) in order to obtain different measurements of pulsatility (one for each beat), which were taken and then averaged.

### 2.3. Pulse Pressure

The pressure in the aorta is different from the values of systolic and diastolic pressure, which can be easily measured from the upper arm using a sphygmomanometer. Indeed, the pressure wave propagating from the center to the periphery (direct wave) encounters the pressure wave generated at sites of bifurcations of arteries and terminal arterioles (reflected wave) and overlaps with it. This phenomenon, known as amplification, makes the pressure variation in the aorta lower than that measured at the brachial site [19].

In the absence of direct invasive measurement of the local pulse pressure in the abdominal aorta, an estimate of the central pressure was obtained by tonometry for subsequent use.

Different calibrations of the pressure curve were performed depending on the instrument used:

- Using the PulsePen, the central pressure was derived from carotid pressure waves, which have been shown to be similar to those recorded in the ascending aorta [20].
- With the SphygmoCor, tonometry was performed at the level of the radial artery and aortic pressure was derived using a validated transfer function [21,22].

### 2.4. Bramwell–Hill Equation

The Bramwell–Hill equation provides the relationship between PWV, pulsatility, and pressure variation [8]:

(3) $$PWV_{est}=\sqrt{\frac{A}{\rho }\frac{\Delta P}{\Delta A}}=\sqrt{\frac{\Delta P}{\rho }\frac{A}{\Delta A}}$$

where:

- ρ represents the density of the blood;
- *A* is the mean area of the blood vessel;
- ΔA is the difference between the maximum and minimum area of the blood vessel during a cardiac cycle;
- ΔP is the difference between the central systolic and diastolic pressures;
- $PWV_{est}$ is the estimated PWV obtained from the measurement of pulsatility (ΔA/A) and pressure variation (ΔP).

For the blood density, we considered (1051 kg/m${}^{3}$); the sensitivity to variations of this parameter is fairly small, as shown in the Results section.

The estimate of PWV provided by Equation (3) was compared with the measured PWV.

Then, we repeated the same for the other two variables, i.e., pulsatility and systo-diastolic pressure difference. Specifically, with respect to pulsatility, the algorithm used to segmented the aorta from the ultrasound videos allowed pulsatility to be measured directly. By inverting the Bramwell–Hill formula, it can instead be computed from the PWV value derived from tonometry and ΔP, allowing for an indirect estimate, as follows:

(4) $${\left(\frac{\Delta A}{A}\right)}_{est}=\frac{\Delta P}{\rho {\left(PWV\right)}^{2}}$$

This estimation was then compared with the measurement on the ultrasound scans.

Finally, by inverting the Bramwell–Hill formula again, we can obtain an explicit relation for the pressure variation as well, using the measured values of PWV and pulsatility:

(5) $${\left(\Delta P\right)}_{est}=\rho {\left(PWV\right)}^{2}\frac{\Delta A}{A}$$

which can then be compared with the value obtained by tonometry.

### 2.5. Experimental Data

Two clinical departments, located in Trieste and Pisa, were involved in this study. The Hospital of Cattinara, Health company Giuliano Isontina (Trieste), recruited seven subjects, two males and five females, with ages varying between 27 to 30 years (mean ± SD 27.98 ± 0.97 years, median 27.89), taking two measurements of PWV for each with the PulsePen. The University of Pisa (Department of Clinical and Experimental Medicine) investigated fourteen subjects, nine males and five females, with ages within a range 25 to 63 years, (mean ± SD 35.79 ± 11.02 years, median 30), taking a single measurement for each using the SphygmoCor. Each subject was monitored once by taking an ultrasound scan of 10 s duration with the system indicated in Section 2.2.

All subjects were healthy volunteers selected among the medical and paramedical staff of the clinical units.

### 2.6. Statistical Analysis

Statistical analyses and all processing were performed using MATLAB® version 9.9.0.1467703, R2020b; Natick, Massachusetts, The MathWorks Inc. (Natick, MA, USA).

In order to compare data obtained by direct and indirect measurement techniques, scatter and Bland–Altman plots were used. The Bland–Altman plots allowed us to evaluate whether two different measurement methods were interchangeable. It was applied for the comparison of measured and estimated values of PWV, pulsatility, and ΔP. The variability was determined as bias ± 1.96 SD according to 95% limits of agreement [23].

## 3. Results

**Figure 1:**
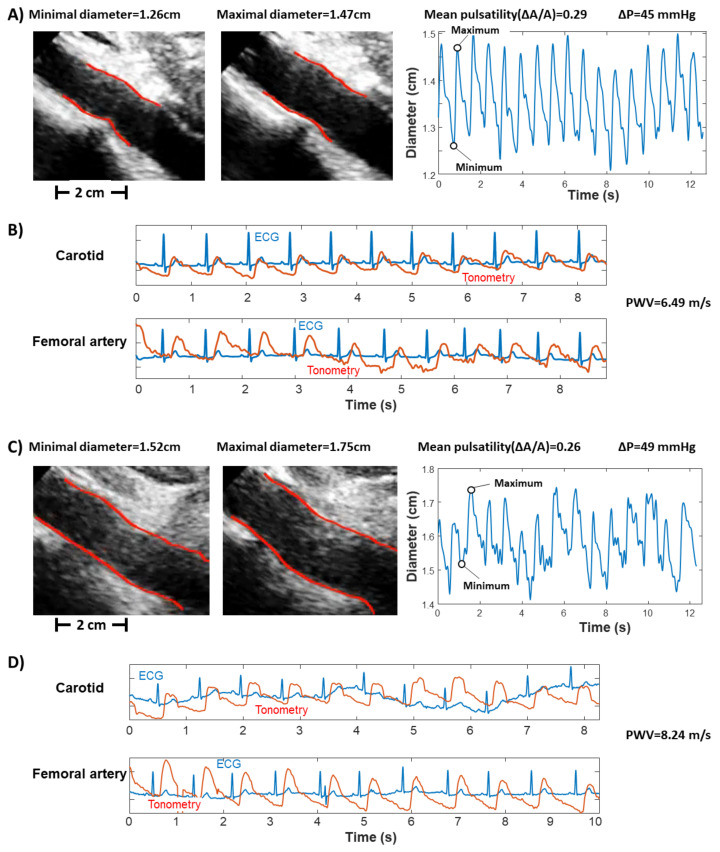
Experimental data from two subjects with different carotid–femoral pulse wave velocities (cf-PWV): (**A**,**B**) subject with medium PWV; (**C**,**D**) subject with high PWV. Ultrasound scans are considered in (**A**,**C**), showing two frames corresponding to local minimum and maximum average diameter (left) and the diameter over time (right). The indication of average pulsatility ΔA/A and pressure variation (ΔP, obtained using PulsePen in these cases) are provided. Tonometry is shown in (**B**,**D**), for both the carotid and femoral artery, with the measured cf-PWV indication.

A few examples of experimental data are shown in Figure 1. Two subjects are considered, one with low aortic stiffness and one with intermediate-high aortic stiffness. Our measurements indicate that the first has smaller cf-PWV, which corresponds to a lower pulsatility with smaller pressure difference; the latter has larger cf-PWV, and higher pulsatility and pressure variation.

**Figure 2:**
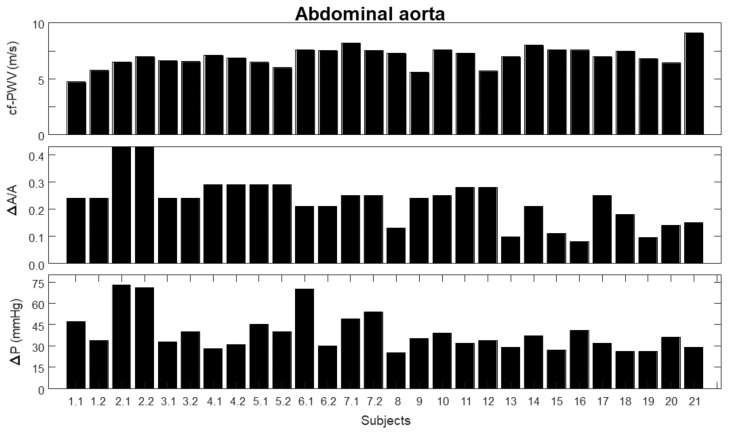
Experimental measurements, including repetitions for specific subjects: carotid–femoral pulse wave velocity (cf-PWV); pulsatility (ΔA/A), indicated as the ratio between the range and the mean value of the area of the aorta measured by automated edge tracking from ultrasound scans; and pressure variation using either PulsePen or SphygmoCor (ΔP).

Figure 2 shows the measurements considered in this study. For the first seven subjects two repetitions each were considered, indicated by the number of the subject followed by either 1 or 2, referring to the two trials. For the other subjects, single measurements were taken; these are indicated by a number. The same numbers are used to indicate the same subjects and trials in the following figures. The following measurements are considered:

1. carotid–femoral pulse wave velocity (cf-PWV);
2. pulsatility (ΔA/A), indicated as the ratio between the range (i.e., maximum minus minimum value) and the mean value of the area of a portion of the abdominal aorta measured by automated edge tracking from ultrasound scans;
3. pressure variation (ΔP), measured using either PulsePen (first seven subjects) or SphygmoCor (remaining subjects).

**Figure 3:**
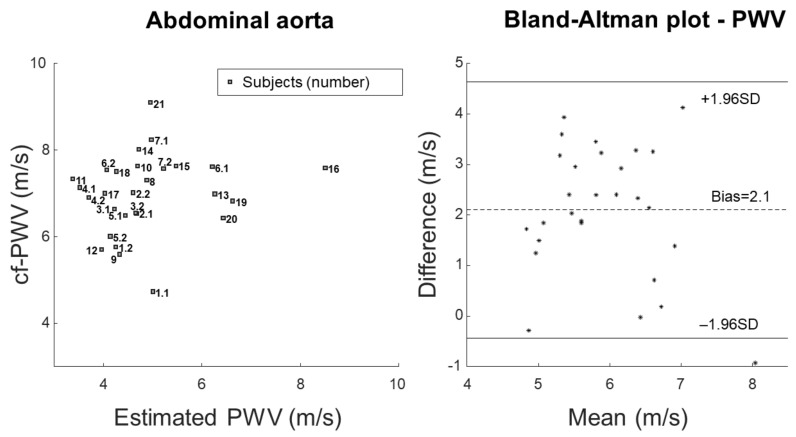
Comparison of PWV measured estimated by the Bramwell–Hill equation using the measurements of pulsatility and pressure variation (correlation coefficient 17.8%). Scatter plot (**left**) and Bland–Altman representation (**right**).

Figure 3 shows the comparison of measured and estimated cf-PWV. The measurement is the same as reported in Figure 2. The estimation was obtained by applying the Bramwell–Hill Equation (3) to the measurements of pulsatility and pressure variation. Scatter plot and Bland–Altman representations are shown on the left and right, respectively.

**Figure 4:**
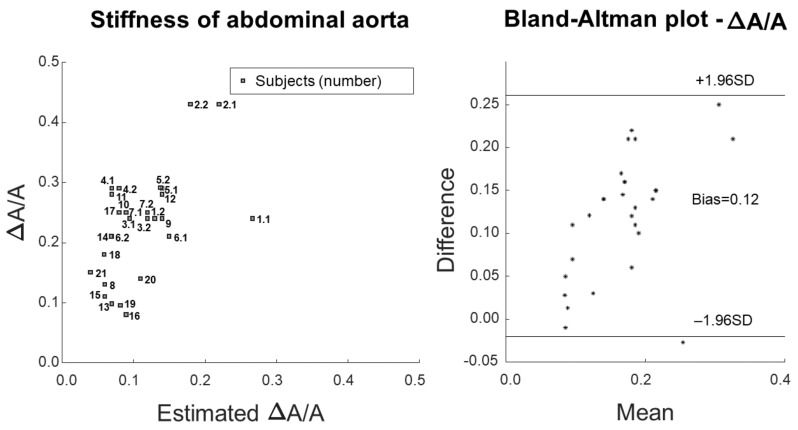
Comparison of measured and estimatedpulsatility by inverting Bramwell–Hill equation and using the measurements of PWV and pressure variation (correlation coefficient 56.1%). Scatter plot (**left**) and Bland–Altman representation (**right**).

Figure 4 shows the comparison of measured or estimated pulsatility, with a scatter plot and a Bland–Altman representation on the left and right, respectively. The measurement is the same as reported in Figure 2, whereas the estimation was obtained by applying Equation (4) (i.e., the inverse of the Bramwell–Hill equation) in order to obtain an explicit expression of the pulsatility from the measurements of cf-PWV and pressure variation.

**Figure 5:**
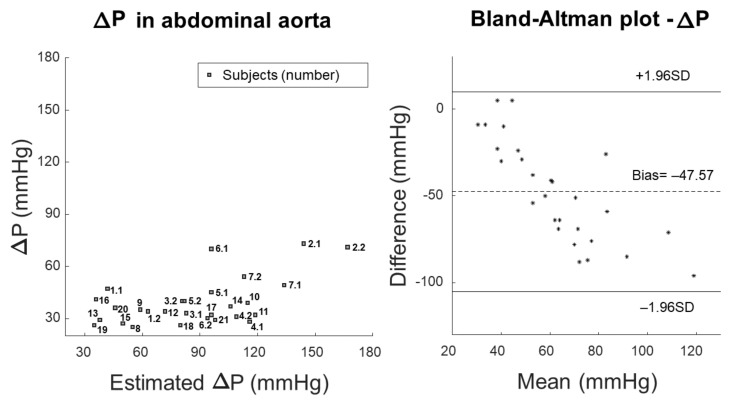
Comparison of pressure variation either measured or estimated by inverting Bramwell-Hill equation and using the measurements of PWV and pulsatility (correlation coefficient 54.5%). Scatter plot (**left**) and Bland-Altman representation (**right**).

Figure 5 shows the comparison of measured and estimated pressure variation using scatter and Bland–Altman plots. The measurement of pressure variation is the same as reported in Figure 2, whereas the estimation was obtained by applying Equation (5) (i.e., the inverse of Bramwell–Hill equation) to obtain an explicit expression for the pressure variation from the measurements of cf-PWV and pulsatility.

**Figure 6:**
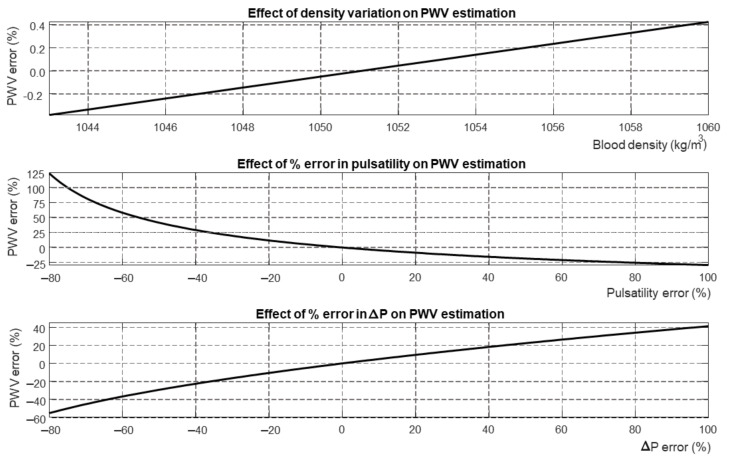
Sensitivity of PWV estimated by Bramwell–Hill equation to certain variables: blood density, error in measuring pulsatility, error in the estimation of pressure variation.

Figure 6 shows a sensitivity analysis of the Bramwell–Hill equation. Different parameters (i.e., blood density, pulsatility, and pressure variation) were varied with respect to a reference, showing the percentage variation of estimated PWV.

## 4. Discussion

Aortic stiffness is an important clinical parameter for cardiovascular evaluation, however, the methodology for its estimation represents an open issue. The use of the Bramwell–Hill equation, which relates PWV to arterial stiffness, allows us to use the non-invasive measurement of PWV to investigate possible pathological states in the cardiovascular system occurring as a result og aging or various pathologies [2]. Stiffness is the ratio between stress and strain in the vessel wall, and is related to the pressure jump and its relative deformation. Arterial systo-diastolic pressure differences can be estimated by validated non-invasive methods [21,22], whereas the deformation can be measured locally by ultrasound scans of a section of the artery. However, the validity of the Bramwell–Hill equation is based on many assumptions, e.g., blood is considered as a Newtonian fluid, the Navier–Stokes equations describing its fluid dynamics can be linearized, and the artery is assumed to be a linear compliant vessel (i.e., the variations of cross-sections are proportional to the pressure jump). Moreover, it is necessary to know the density of the blood, which it is seldom measured in clinical practice. Therefore, it makes sense to question the validity of this equation when applied to experimental data on account of its sensitivity to the various contributions involved (i.e., pressure jump, pulsatility, blood density), which can only be estimated with uncertainty.

### 4.1. Outcomes

We used state-of-the-art methods to measure the cf-PWV, the pulsatility of a portion of the abdominal tract of the aorta, and the central aortic pressure jump. Then, we tested the validity of the Bramwell–Hill equation when estimating one of the three variables while knowing the other two, assuming a constant value of blood density taken from the literature.

Our results indicate that there is important indeterminacy in estimating one variable from measurements of the other two, as this results in bias and large variations of all estimations. This is not due to the assumed value of blood density, as PWV does not change much when varying it across the range of values reported in the literature (Figure 6). The mistakes could be partially interpreted in light of the estimation of PWV by the Bramwell–Hill equation being quite sensitive to errors in the measurement of the pressure jump and pulsatility (Figure 6).

A mistake in the estimation of the pressure jump was expected, as the transfer function we used was validated to estimate the central pressure in the ascending aorta, not in the abdominal track, where it may be larger due to the pressure amplification phenomenon. However, when estimating the pressure jumps needed to account for the measured PWV and pulsatility, the obtained values were often not reliable (Figure 5).

On the other hand, any mistake in estimating pulsatility was not expected to be large, as the automated edge identifications were visually inspected and large errors (i.e., greater than 10%) excluded.

A further observation is that cf-PWV accounts for the average velocity of pulses along a portion of aorta, whereas the ultrasound scan was localized in a specific cross-section. Previous studies have documented that local and regional measures of PWV are not interchangeable, and that aortic PWV seems to decrease along the aortic path [24]. In general, our results agree in identifying a large difference of up to 2 m/s between the local PWV in the aorta and the cf-PWV.

However, previous studies failed to identify the reasons for this discrepancy, which may be attributable to multiple factors, such as the measurements (or physiological variations) of cf-PWV, evident mistakes in the pressure jump, and poor reliability of the Bramwell–Hill equation due to the many approximations involved.

### 4.2. Significance

The evaluation of aortic stiffness is a relevant clinical parameter that has been widely validated as an independent predictor of cardiovascular events [25]. Although cf-PWV has been proposed as the gold standard method for the evaluation of aortic stiffness [5,6], there are questions as to the possibility of using it practically due to the need for dedicated equipment and experienced operators. Several other methods have been proposed to quantify the stiffness of large arteries, and the use of ultrasound to evaluate this parameter is particularly attractive on account of the wide diffusion of echography machines and technological advancements that allow high-quality imaging acquisition. Thus, examining aortic stiffness with an ultrasound device may have relevant clinical uses. Previous studies evaluating the relationship between cf-PWV and arterial stiffness with ultrasounds focused on the carotid artery, a segment that is close to the aorta and relatively easy to examine with the current ultrasound equipment [26,27,28,29]. To the best of our knowledge, our study is the first to compare cf-PWV to local aortic PWV determined using the Bramwell–Hill equation and ultrasound imaging. Both of these methods are non-invasive, relatively inexpensive, and easily applicable in patient care. However, estimating possible sources of bias in this comparison is important in order to establish a correct methodological approach and to use local aortic PWV values in clinics.

### 4.3. Limitations

We have already mentioned factors that may affect the comparison between cf-PWV and local aortic PWV, namely, possible indeterminacy in the measurements or physiological variations of cf-PWV, evident mistakes in the pressure jump, and poor reliability of the Bramwell–Hill equation due to the many approximations involved. Moreover, cf-PWV and local aortic PWV are an integral and a local measurement, respectively, as the first depends on the average velocity of the pulse along an entire portion of aorta, while the second accounts for only a specific section visualized by the ultrasound scan. In addition, in the present investigation we used two different tools to measure cf-PWV and to estimate aortic PWV (Sphygmocor and PulsePen devices), which may have introduced a further methodological discrepancy. Moreover, the limited number of observations, which were conducted only in healthy subjects, may limit the generalization of our results to the overt pathological states that are the main outcome for the clinical use of arterial stiffness measurements. Furthermore, there are patients for whom the investigation of abdominal aorta by ultrasound could be critical (e.g., those with obesity or a large amount of bowel gas), in which case the generalization of our approach could be difficult.

### 4.4. Future Perspectives

While cf-PWV has been widely validated in several studies, further methodological improvements are required for the estimation of aortic PWV using the Bramwell–Hill equation. On the one hand, the automation of image processing for the correct quantification of areas and pulsatility is a mandatory step for implementation of this method with the current ultrasound devices. On the other hand, large studies to establish reference values for local aortic PWV as well as clinical validation of the effect of this method on health outcomes are both needed before it can be applied in clinical practice.

## 5. Conclusions and Further Work

Our study compared two methods (cf-PWV and ultrasound-derived aortic PWV) which are notably correlated by the Bramwell–Hill equation, identified several relevant discrepancies, and quantified the possible sources of bias inherent to the equation. Our results suggest that these two measures should not be considered merely interchangeable, and that the bias between them is mainly attributable to issues in blood pressure estimation. It is possible that measuring cf-PWV by tonometry and aorta pulsatility by ultrasound scan could be important in further improving the clinical picture.

Additional studies are needed in order to identify the relationship between cf-PWV and ultrasound-derived aortic PWV in both healthy and pathological states. The identification and possible correction of the sources of bias identified in this study may lead to methodological improvements and the validation of easily accessible tools for cardiovascular medicine that are reliably applicable in patient care.

## Data Availability

Data are available from the corresponding author upon reasonable request.

## Abbreviations

The following abbreviations are used in this manuscript:

cf-PWV	carotid–femoral Pulse Wave Velocity
PWV	Pulse Wave Velocity
